# Separate Courts of Justice for Children

**Published:** 1904-11-12

**Authors:** 


### SEPARATE COURTS OF JUSTICE FOR CHILDREN.

The outcome of the Conference convened last week by the Committee on Wage-Earning Children, on the subject of separate courts of justice for children, was a recommendation that any legislation to be promoted should make provision, as a compulsory measure, for the treatment of children who are now liable to be brought to the police court, in places separate and apart from the court for adult cases. This would seem to imply that such cases should be considered, not necessarily in a separate building, but in a separate room, or at a different time from the adult cases, as has recently been arranged in Ireland. In Dublin, for instance, children's cases will in future be considered in a room in the police-court known as the Central Hall, by one of the divisional magistrates, and while waiting for their cases to come on, the children will be kept in a special waiting-room. In Cork the magistrates will make arrangements for the hearing of children's cases on a particular day, or at some fixed hour on any day, the juveniles and their parents alone being present. In Massachusetts and some other States, Canada and South Australia, children's courts have long been an accomplished fact, that of Massachusetts dating as far back as 1863, and the recent development in Ireland, Mr. Albert Spicer, of the State Children's Association, pointed out, although not everything that is desired by the promoters of separate courts, is a step in the right direction. The Conference further agreed that it was desirable to make Section 4 (1) of the Youthful Offenders Act of 1901 obligatory, and to make the remand homes available for the period between arrest and appearance in court. The section referred to provides for the establishment of remand homes to which children awaiting trial may be sent. It is, however, optional, and only comes into operation on remanding or committing for trial. The question as to whether legislation for the establishment of separate courts was necessary or not was not voted upon, but so far as London is concerned (and a considerable portion of the time at the disposal of the Conference was devoted to the consideration of the question as it affects the metropolis), it was decided to ask the Home Secretary to re-introduce the Justices Jurisdiction Bill (London), which enables separate courts to be set up by Order in Council, on the recommendation of the Home Secretary, on application to the justices; provides payment for the clerk and other officers; sets out certain matters in the schedule which the courts may touch, but provides for the list being varied by Order in Council; provides against overlapping jurisdiction, and secures uniformity in fees, etc. It was suggested that if the Bill were re-introduced, clauses dealing with other parts of England might be added, together with provision regarding probationary officers. This Bill, it will be remembered, was introduced too late in the session of 1901 to become law, and it has not been re-introduced. It refers only to London, and is not confined to children's cases. But it is based upon the careful and prolonged deliberations of an influential committee of inquiry. It was prepared and introduced by the Home Office, and passed by the House of Lords. It is, therefore, a sound basis for new legislation.

